# Gold Nanoparticle Size-Dependent Enhanced Chemiluminescence for Ultra-Sensitive Haptoglobin Biomarker Detection

**DOI:** 10.3390/biom9080372

**Published:** 2019-08-14

**Authors:** Narsingh R. Nirala, Giorgi Shtenberg

**Affiliations:** Institute of Agricultural Engineering, ARO, the Volcani Center, Bet Dagan 50250, Israel

**Keywords:** enhanced chemiluminescence, gold nanoparticles, haptoglobin, mastitis

## Abstract

Bovine mastitis (BM) is a frequent disease in the dairy industry that causes staggering economical losses due to decreased milk production and increased health care costs. Traditionally, BM detection depends on the efficacy and reliability of analytical techniques that measure somatic cell counts (SCC), detect pathogens, and reveal inflammatory status. Herein, we demonstrate the detection of bovine haptoglobin, a well-documented acute phase protein for evaluating BM clinical status, by utilizing hemoglobin-binding capacity within luminol chemiluminescence (CL) system. The resulting haptoglobin–hemoglobin complex reduces the CL signal proportionally to inherent haptoglobin concentrations. Different sizes of cross-linked gold nanoparticles (GNPs) were examined for enhanced CL (eCL) signal amplification, presenting over 30-fold emitted radiation enhancement for optimized size within real milk samples with respect to nanoparticle-free assay. The eCL values were proportionally related to nanoparticle size and content, influenced by SCC and pathogen type (e.g., *Escherichia coli* and coagulase-negative staphylococci). The optimized bioassay showed a broad linear response (1 pg mL^−1^–10 µg mL^−1^) and minute detection limit of 0.19 pg mL^−1^, while presenting quantitative performance in agreement with commercial ELISA kit. Finally, the resulting optimized eCL concept offers an efficient label-free detection of haptoglobin biomarker, offering means to diagnose the severity of the associated diseases.

## 1. Introduction

Chemiluminescence (CL) is widely applied chemically driven electromagnetic radiation for real-life applications, from clinical monitoring, food and pharmaceutical safety control, and up to trace levels of environmental pollutants analysis, utilizing its high sensitivity toward the assessment of the limiting target reactant involved in the luminol reaction [1,2,3,4,5,6,7,8,9,10,11]. The low emitted radiation within complex samples such as blood, milk, urine, or biological tissues, can be efficiently augmented by the addition of a catalyst, e.g., metal or semiconductor nanoparticles, catalytic ions, enzymes, into the luminol CL system. Gold nanoparticles (GNPs) are one of the most frequently used nanoparticles for analytical applications harnessing the ease of opto-electronic property fine-tuning by mild changes in the nanoparticles size, morphology, surface functionality, and local environment [12,13]. Indeed, numerous case studies and reports employed GNPs for enhanced CL (eCL) presenting a repertoire of novel ultrasensitive bioassays [4,5,6,12,13,14,15,16,17,18,19]. Singh et al. have reported the use of sequence-specific single-stranded DNA probes anchored on the exterior of GNPs surface for the hybridization with the complementary DNA target regions of *Ceratocystis fagacearum* [15]. The molecular recognition of the target-specific hybridization resulted in instantaneous GNPs aggregation into cluster formation, which directly induced an eCL signal. The label-free strategy has presented a limit of detection (LOD) of 260 fM, two orders of magnitude higher than plasmonic colorimetric and absorption spectrometry of single GNPs. A similar hybridization concept was used for quantitative analysis of thrombin found in patient’s serum for clinical studies utilization [18]. The authors reported CL amplification based on multi-DNAzymes-functionalized GNPs performed in multichannel microchip with amplified reaction efficiency and reduced analysis time. Lyu et al. have fabricated a low-cost and miniaturized electro-CL bio-platform for the detection of discharged hydrogen peroxide by macrophage cells and for the measurement of antioxidant activity for novel drug development process [20]. The bio-platform allowed synchronized analysis of six samples within 1 min of reaction. A linear correlation over the range of 100 nM–200 μM and LOD of 25.3 nM was shown. The bio-platform can be adapted for the design of upgraded biosensing devices, designing novel lab-on-a-chip architectures, and nano-fluidic schemes. Bui et al. have reported on a competitive CL detection assay based on tris(2-carboxyethyl)phosphine (reducing agent) interaction with both microorganisms and GNPs, which was used for rapid microbial screening and identification in less than 10 min, while presenting a LOD as low as 100 cfu mL^−1^ and a proportional response up until 10^7^ cfu mL^−1^ [4]. Moreover, the concept was demonstrated for real-life samples in less than 1 h through the specific identification of methicillin-resistant *Staphylococcus aureus* in urine and environmental samples enclosing a complex mixture of bacteria.

In the current study, we show efficient liquid-phase CL system for haptoglobin detection and correlating the optical response to dairy cattle health status. Specifically, bovine mastitis (BM) is a frequent disease in the dairy industry that causes staggering economical losses due to decreased milk production and increased health care costs [21]. Traditionally, BM detection depends on the efficacy and reliability of analytical techniques that are designed to measure somatic cell counts (SCC), detect causative pathogens, reveal inflammation, or evaluate the disease onset with the associated biomarkers [22]. The last factor includes a number of acute phase proteins found in serum that are secreted into milk upon inflammation, infection, or trauma and are used as predicting analytical biomarkers for clinical status assessment [21,23]. Haptoglobin is a well-documented acute phase protein induced by various cattle diseases presenting elevated concentrations (up to 100-fold) in comparison to healthy conditions. Haptoglobin is generally distributed upon damage or leakage in mammary vascular cells, hence accumulating in milk [24]. There are several commercially available immunoassays, such as ELISA or single radial immunodiffusion for the detection of haptoglobin that utilize hemoglobin-binding capacity, which all suffer from cost of a measurement and elongated and labor-intensive protocols [22,23].

**Figure 1:**
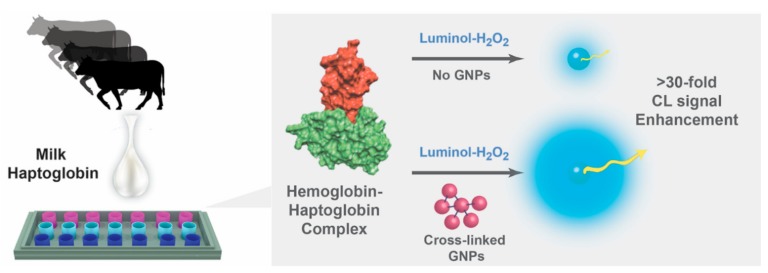
A schematic illustration of the haptoglobin sensing concept using cross-linked gold nanoparticles (GNPs) for enhanced chemiluminescence (eCL). The hemoglobin-modified plate specifically reacts with inherent haptoglobin content in milk. The subsequent protein–protein (hemoglobin–haptoglobin) complex hinders the catalytic activity of hemoglobin in proportion to inherent haptoglobin concentration. Reduced quantity of target molecules can be amplified by the addition of cross-linked GNPs onto the luminol mixture to intensify the emitted radiation for highly sensitive detection of the acute phase molecules.

Herein, we have applied a similar sensing concept for sensitive detection of bovine milk haptoglobin content based on liquid-phase luminol-hydrogen peroxide (H_2_O_2_) system, as schematically illustrated in Figure 1. Different sizes of cross-linked GNPs were fabricated for the study and evaluated under optimized conditions for their catalytic activity, as previously published [16]. The maximal eCL effect for optimal particle size and content was evaluated with respect to the specific hemoglobin–haptoglobin complex formation after the application of the studied milk samples. The resulting complex inhibited the catalytic activity of hemoglobin within the luminol system and reduced the CL signal in proportion to inherent haptoglobin concentration. Additionally, the SCC levels and the causing pathogenic bacteria influence on the haptoglobin concentrations were evaluated with respect to the sensing performance of a commercial ELISA kit.

## 2. Materials and Methods

### 2.1. Chemicals

Gelatin, glutaric dialdehyde (50 wt.%), bovine hemoglobin, luminol sodium salt, bovine haptoglobin, sodium borohydride, sodium hydroxide, 1,3-propanedithiol (PDT), H_2_O_2_, methanol, and analytical buffers were acquired from Sigma-Aldrich (Saint Louis, MA, USA). Trisodium citrate dihydrate and tetrachloroauric(III) acid were purchased from Merck (Darmstadt, Germany). Specific ELISA Kit for haptoglobin detection was purchased from Abcam (Cambridge, UK) (catalog # ab137977).

### 2.2. Synthesis of Different Sizes of GNPs

Several sizes of GNPs (2.6, 8, 13, 25, and 38 nm) were synthesized by a modified procedure of Brown et al. [12,25]. Briefly, for 2.6 nm, 25 mL of tetrachloroauric(III) acid (0.01% *w*/*w*) solution was rapidly added with 0.25 mL of 1% trisodium citrate and 0.25 mL of 0.075% ice cold sodium borohydride under robust stirring. The solution was stirred at room temperature for 0.5 h to attain the desired nanoparticles size and refrigerated until further use. A similar procedure was used to obtain other nanoparticle sizes by varying trisodium citrate volume (1.0, 1.4, 0.375, and 0.125 mL for the preparation of 8, 13, 25, and 38 nm GNPs, respectively). Stock solutions for all GNPs sizes were normalized to 2.36 mg mL^−1^ before any subsequent analysis or treatment. Finally, the resulting nanoparticles were cross-linked for 10 min in the presence of 5 mM PDT (cross-linked GNPs) in methanol. Due to the time dependent cross-linking process with PDT, freshly cross-linked GNPs were prepared before each experiment.

### 2.3. CL Assay

The effect of GNPs size on CL signal constancy was evaluated at optimized reactant concentrations (1 µg mL^−1^ of hemoglobin-modified plate, 0.45 mM luminol sodium salt, 0.01 mM H_2_O_2_, 15 mM sodium hydroxide, pH ~ 12), as previously reported by Nirala et al. [16]. Briefly, a 96-well plate was treated with 10 mg mL^−1^ gelatin in 0.05 M carbonate buffer (pH 9.6) for 2 h, followed by vigorous rinsing with 0.05 M phosphate buffer saline (PBS) pH 7.4 and two consecutive rinses with ultrapure water. Next, the well plate was modified with 2.5 wt.% glutaraldehyde for cross-linking 1 µg mL^−1^ hemoglobin, followed by a thorough cleaning process [16]. CL enhancement measurements of the different sizes of cross-linked GNPs dilutions (stock solution, 1.25, 1.66, 2.5, 5, 10-fold, and a control—without the addition GNPs) were achieved by adding 20 µL of diluted GNPs onto 100 µL of 10-fold diluted luminol solution followed by immediate CL value measurements [16]. The data are presented as eCL factor of a specific diameter (*d*(*x*)) of cross-linked GNPs and defined as:

(1) $$eCL\;Factor_{GNPs\;d\left(x\right)}=\frac{CL\;Intensity_{GNPs\;d\left(x\right)}}{CL\;Intensity_{without\;GNPs}}$$

where $CL\;Intensity_{GNPs\;d\left(x\right)}$ is the CL value in the presence of cross-linked GNPs size *d*(*x*) and $CL\;Intensity_{without\;GNPs}$ is the CL value obtained while omitting the catalysts from the luminol solution, while using milk-free conditions (maximal CL values).

### 2.4. Milk Sample Collection and Preparation

Samples were collected by trained personnel from specific quarters of Holstein cows (Volcani Center) representing healthy, spontaneous subclinical, or clinical mastitis, as previously described [16].

### 2.5. CL Assay Calibration

Reference bovine haptoglobin stock solution was diluted to a range of concentrations (from 0 to 10 µg mL^−1^). A hundred microliters of each haptoglobin concentration was applied on the plate assay for 0.5 h, followed by washing procedures (PBS and two consecutive ultrapure water rinsing steps). Next, milk samples with insignificant haptoglobin concentrations (100 µL) were added to each well, incubated for 0.5 h, and thoroughly rinsed. The modified plate CL values were immediately measured after the addition of 20 µL of optimal cross-linked GNPs (both size and dilution-fold) merged with 100 µL of 10-fold diluted luminol solution.

### 2.6. Haptoglobin Quantification

Defatted milk samples were received by short centrifugation (2000× *g* for 10 min) followed by twenty-fold dilution with ultrapure water. Haptoglobin concentrations above the calibration curve linearity range were additionally diluted. Subsequently, 100 µL of analyzed sample was applied for 0.5 h followed by a thorough washing procedure to eliminate any unbound interfering molecules. Lastly, cross-linked GNPs and diluted luminol solution (20 and 100 µL, respectively) were immediately used for CL value assessment. The enhanced CL factor was calculated with respect to the obtained CL values in the absence of cross-linked GNPs for optimal nanoparticle size, while using milk-free conditions as a control (maximal CL values). Haptoglobin concentrations were calculated based on the calibration curve and were compared to the values obtained from a commercial ELISA Kit.

### 2.7. Instrumentation

Extinction spectra were recorded with a multimode microplate reader, Varioskan™ LUX by Thermo Scientific (Waltham, MA, USA), characterized by ±0.003 Abs units for accuracy, standard deviation <0.001 Abs units for precision, and wavelength resolution of 1 nm. The CL intensity values were acquired with the same reader by monitoring peak intensity. Dynamic light scattering (DLS) was used to analyze the hydrodynamic diameter of the different GNPs using a Zetasizer Nano ZS, (Malvern, UK) while performing the measurements at 25.0 °C. The results were obtained from intensity-based distributions using maximal peak position. Transmission electron microscopy (TEM), JEOL JEM-1400, was applied for studying structural morphology and size, operated at 120 kV.

### 2.8. Statistical Analysis

Statistical analysis was performed using a Student’s t-test with a minimum confidence level of 0.05 for statistical significance and assuming unequal sample sizes and unequal variance. All values are reported as the mean ± standard deviation (*n* ≥ 3), unless otherwise stated.

## 3. Results and Discussion

### 3.1. GNP Characterization

**Figure 2:**
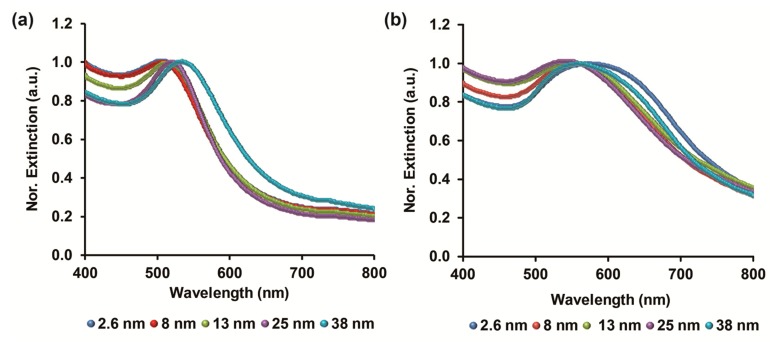
Normalized UV–VIS extinction spectra of the different GNPs sizes: (**a**) Before and (**b**) after PDT cross-linking.

Different sizes of GNPs were produced according to a modified procedure of Brown et al. [12,25]. Figure 2a shows the normalized UV–VIS extinction spectra of the resulting nanoparticle sizes, in which a characteristic surface plasmon resonance (SPR) peak was obtained. The colloidal GNPs showed a correlative SPR peak position which red-shifted with nanoparticle size enhancement (505, 507, 519, 522, and 534 nm for 2.6, 8, 13, 25, and 38 nm, respectively) [13,17]. Subsequently, the GNPs were aggregated via PDT cross-linking, by exploiting its high affinity toward thiol groups on the exterior of nanoparticles surfaces. The superclusters formation is presented by the longer wavelength resonances within the range of 540–580 nm that are red-shifted correspondingly to clusters size growth, Figure 2b [26]. The SPR resonance of 2.6, 8, 13, 25, and 38 nm cross-linked GNPs are located at 572, 542, 551, 555, and 560 nm, respectively, and are ascribed to the degree of capping with dithiol molecules and regrouping of the formed clusters [17,27]. It should be noted that conventional GNPs cross-linking will result at higher SPR red-shift; however, in our case a short period of 10 min was applied for sufficient and controlled clusters formation which will potentially intensify the CL signal by catalytically active mass of nanoparticles within the luminol assay [12]. Moreover, the cross-linking process or any GNPs aggregation is a time-dependent reaction, as prolonged process will result in substantial physiochemical interactions and the augmentation of the cluster dimensions [27,28]. Table S1 (Supplementary Materials) summarizes the averaged diameter of the fabricated GNPs before and after PDT cross-linking measured by DLS measurements. Indeed, the obtained data correlated to the planned particle size of unmodified GNPs. However, the opposite trend was shown for cross-linked GNPs in which the decrease in particle diameter resulted in higher collective hydrodynamic assembly. The result is ascribed to the increased surface area of smaller nanoparticles that offer profound anchoring points for dithiol molecules between neighboring particles upon the cross-linking process [29,30]. TEM images of the different nanoparticle dimeters further confirm the UV–VIS and DLS studies, see Figure S1 (Supplementary Materials).

### 3.2. Enhanced CL Signal Optimization

**Figure 3:**
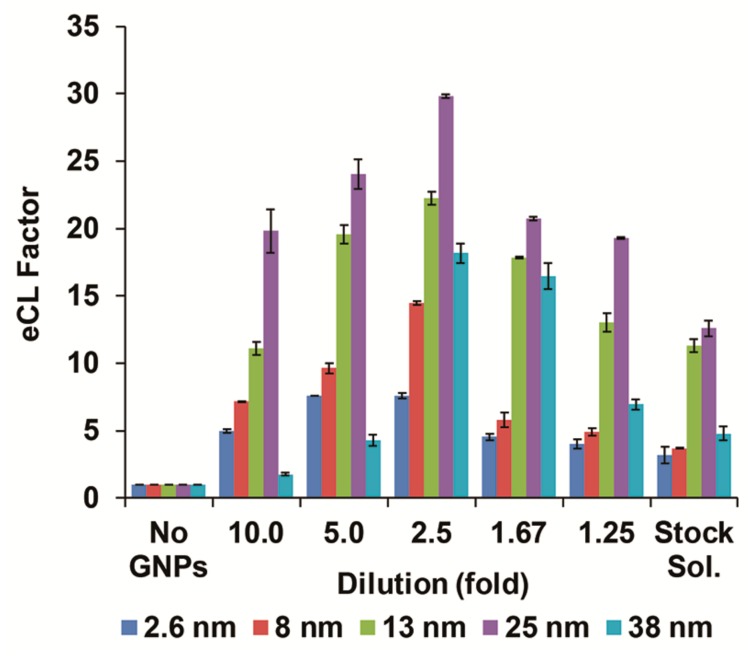
Enhanced CL factor of the different cross-linked GNPs dilutions for the various nanoparticle sizes within the luminol system. The enhancement values were calculated with respect to CL values in the absence of cross-linked GNPs. Data are reported as mean ± standard deviation (*n* ≥ 3).

Colloidal and aggregated GNPs in various sizes and morphologies have been utilized as catalysts for eCL reaction systems depicting extensive enhancement in the emitted radiation in comparison to conventional assays (which omit the use of nanoparticles), while interacting with luminol assay intermediates [1,2,3,5,6,12]. Higher catalyst content within a given mass leads to an escalated rate of catalytic activity ensuing eCL output, while the aggregated form is substantially preferable [12]. Thus, the colloidal GNPs stock solutions were normalized to 2.36 mg mL^−1^ before any treatment (i.e., cross-linking) to control the initial particle content. The enhancement effect of the obtained different sizes on luminol–H_2_O_2_–hemoglobin sensing scheme was studied by assessing the CL radiation. First, different volumes of cross-linked GNPs stock solution (0, 10, 20, 40, 60, 80, and 100 µL) were mixed with ultrapure water to a final volume of 100 µL producing a control (omitting cross-linked GNPs addition), 10, 5, 2.5, 1.67, and 1.25-fold dilutions and the original stock solution (without any dilution), respectively. Next, the CL intensities were evaluated by mixing 20 µL of each set of diluted cross-linked GNPs with 100 µL of 10-fold diluted luminol mixture. Figure 3 shows the eCL factor values of the hemoglobin-modified plate assay optimized conditions for each nanoparticle size as a response to the catalyst content augmentation. The obtained data for each nanoparticle size were normalized with respect to CL values in the absence of cross-linked GNPs. Indeed, the eCL factor was amplified with higher catalyst concentration (involved in the reaction), decreasing the dilution magnitude from 10-fold up to 2.5-fold dilution for all analyzed cross-linked GNPs sizes. Above this point, 1.67, 1.25-fold dilution and stock solution, a similar decrease in CL signals was obtained for all nanoparticles used, which is ascribed to intensified catalytic consumption of the luminol mixture constituents. Moreover, it can be seen that within each dilution set, the eCL factor was proportionally increased with nanoparticle size enlargement up to the optimal size and reduced immediately after, as previously shown [14,31]. This can be accredited to the GNPs size-dependent electron-transfer process that influences the CL reaction [13]. For instance, the highest eCL factor values were obtained for 2.5-fold dilution presenting data of 7.6 ± 0.2, 14.4 ± 0.1, 22.2 ± 0.5, 29.8 ± 0.1, and 18.1 ± 0.7 for 2.6, 8, 13, 25, and 38 nm of cross-linked GNPs, respectively. The mechanism behind luminol reaction catalytic activity is attributed to the metal nanoparticles’ surface charge properties [32]. Conventional citrate-reduced GNPs possess a negative charge that electrostatically repulses both anionic luminol and hydroperoxide ions, thus significantly reducing the overall CL signal [16]. However, herein a combined borohydride–citrate reduction was applied for gaining less negative surface charge on the produced GNPs [12]. Moreover, cross-linking with PDT decrease the overall negative surface charge density in respect to non-aggregated GNPs, hence preferring the adsorption between the aggregated clusters and the anionic luminol mixture constituents [33]. Taking into account the eCL factor and the overall reagents depletion, the optimized settings for luminol–H_2_O_2_–hemoglobin system were set to 2.5-fold cross-linked GNPs dilution for all sizes.

**Figure 4:**
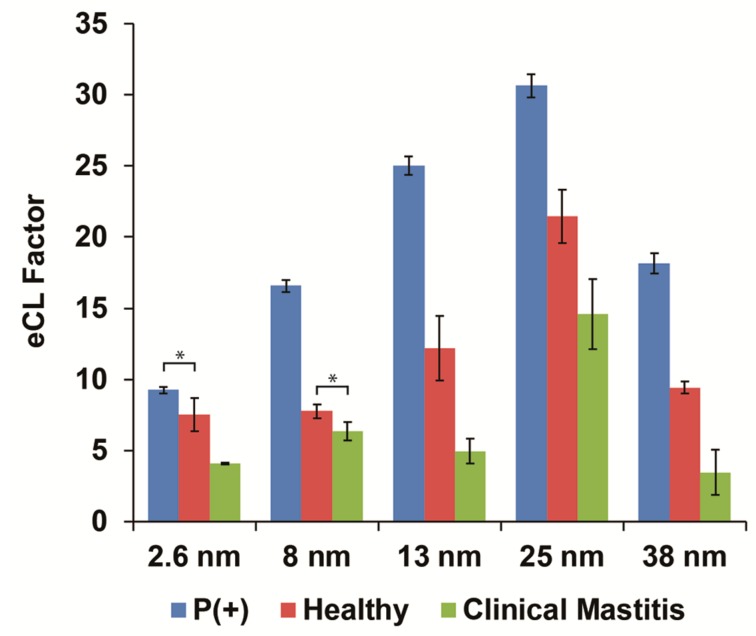
Enhanced CL factor of the various cross-linked GNPs sizes for luminol system within healthy and mastitic milk samples and a positive control (omitting milk from the assay). The enhancement values were calculated with respect to CL values in the absence of cross-linked GNPs, while omitting milk from the assay. Data are reported as mean ± standard deviation (*n* ≥ 3). * Significantly different (t-test, *p* < 0.05).

Next, based on the obtained correlation between maximal eCL factor and cross-linked GNPs size within the luminol–H_2_O_2_–hemoglobin CL system, we investigated the compatibility of the assay toward dissimilar milk quality differentiation and potentially deducing the optimal particle size for the performed analysis. Thus, skim milk samples were reacted with the modified wells followed by a vigorous rinsing procedure to remove interfering milk constituents that can inhibit the CL signal. Figure 4 depicts the obtained eCL factor of healthy and mastitic milk samples (boundary conditions for quality estimation and low and high haptoglobin concentrations, respectively) with respect to a positive control (milk-free solution, expecting maximal eCL factor values) for all analyzed cross-linked GNPs sizes. The data were similarly normalized to CL values obtained in the absence of cross-linked GNPs for each nanoparticle size, while omitting milk from the assay. The quality differentiation was based on haptoglobin content within each sample and its influence on the inhibition of the catalytic activity of luminol CL system in response to hemoglobin–haptoglobin complex formation. Increased haptoglobin concentration within each sample will produce higher protein–protein (hemoglobin–haptoglobin) complexes, thus limiting the catalytically active species of the modified plate assay, producing reduced CL intensities. Previous studies verified the irreversible protein–protein binding with high specificity and marked changes in heme reactivity [34,35]. Indeed, eCL factor values were reduced significantly (t-test, *p* < 0.05) upon deterioration of milk quality for all particle sizes (e.g., values of 30.6 ± 0.8, 21.4 ± 1.9, and 14.6 ± 2.5 for milk-free assay, healthy, and clinical mastitis, respectively, using 25 nm cross-linked GNPs). It has been previously shown that naturally occurring clinical mastitis in dairy cows suffering from acute phase response increases the inherent haptoglobin values both in serum and milk [21]. The nanoparticle size-dependent correlation toward the eCL factor was similarly shown within real milk samples, presenting intensified values with the elevation in cross-linked GNPs diameter up to optimal size and reduced immediately after. These results further strengthen the specificity of the proposed system toward haptoglobin biomarker. Therefore, based on the obtained results, 25 nm cross-linked GNPs were used for the consecutive analysis within the luminol system for specific haptoglobin detection. It should be mentioned that other particles size (i.e., 13 or 38 nm) could be also used for applicative determination of haptoglobin content in milk samples.

### 3.3. Haptoglobin Quantification Based on eCL Factor

**Figure 5:**
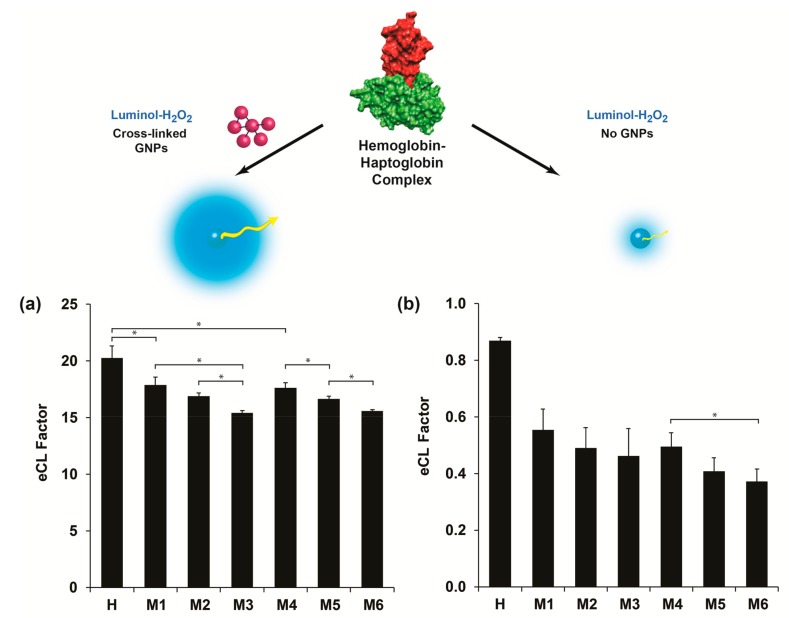
Enhanced CL factor for the analyzed milk samples: (**a**) With and (**b**) in the absence of 25 nm cross-linked GNPs onto the luminol CL system. **Top inset:** A schematic illustration of the intensified emitted radiation (eCL concept). The enhancement values were calculated with respect to CL values in the absence of cross-linked GNPs, while omitting milk from the assay. Data are reported as mean ± standard deviation (*n* ≥ 3). * Significantly different (t-test, *p* < 0.05).

The analytical resolution of the developed plate assay for haptoglobin detection was further investigated for a comprehensive milk quality spectrum. The analyzed milk samples acquired from different animal udders are summarized in Table 1. *Escherichia coli* (*E. coli*) and coagulase-negative staphylococci (CNS), two predominant pathogenic bacteria causing BM at three SCC levels (~300,000, 800,000, and above 1,000,000 cells for subclinical and clinical BM, respectively) were used. Moreover, healthy milk (sample H) was set as a control expecting minute haptoglobin values in which negative microbiological output was obtained (no traces of any contaminants or pathogens) presenting an insignificant SCC value of 60,000. It should be highlighted that sample H was adopted as a representative of other milk samples with a similar SCC value range, haptoglobin content, and negative biological contamination (data not shown). All skim milk samples were reacted with the hemoglobin-modified plate assay according to the noted protocol while using optimized condition, both for catalyst content and size. Figure 5a depicts the eCL factor values of the analyzed milk samples with the addition of 25 nm cross-linked GNPs into the luminol assay. Indeed, the eCL factor was substantially reduced upon SCC level escalation, presenting data of 17.9 ± 0.7, 16.9 ± 0.3, and 15.4 ± 0.2 for M1, M2, and M3 (all positive with *E. coli* bacteria) with respect to sample H (negative control with eCL factor value of 20.3 ± 1.1). The presented data were substantially different (t-test, *p* < 0.05) both within the *E. coli* samples and with respect to the healthy milk. However, one exception of non-significant differentiation was shown between M1 and M2 samples (t-test, *p* > 0.05), which can be ascribed to the mild difference in the SCC values and the severity of the mastitis. A similar eCL factor value trend was obtained for the CNS pathogen (samples M4, M5, M6, t-test, *p* < 0.05), thus suspecting a direct correlation between eCL factor values and haptoglobin concentration within the different milk samples. Samples M3 and M6 identified with clinical mastitis (>1,000,000 SCC score) presented the lowest eCL values assuming higher inherent haptoglobin content. This can be accredited to the severity of BM at higher SCC scores for both pathogens resulting in higher haptoglobin concentrations within the examined milk samples [21,24]. Non-significant differentiation (t-test, *p* > 0.05) between the SCC scores of both pathogens was obtained by omitting the cross-linked GNPs from the CL assay (except between M4 and M6, t-test, *p* < 0.05), Figure 5b. The non-enhanced data can be used for validating the increase in haptoglobin levels, above a threshold cutoff value, thus differentiating between healthy samples and mastitic samples (entire clinical spectrum: Sub, clinical, and chronic), eCL factor values of 0.87 ± 0.01 and 0.47 ± 0.07, respectively. These results further confirm the applicability of extensively enhanced emitted radiation, by at least 30-fold, in comparison to conventional assays (with and without the usage of cross-linked GNPs, respectively), or above 10-fold with our previous proof-of-concept study [16], while interacting with luminol assay intermediates. Additionally, these results effectively discriminate between the obtained milk qualities based on the protein–protein complex formation (specific interaction of two globular protein surfaces to form a tightly bound and irreversible multicomponent composite) and, thus, depicting the severity of the occurring BM within the herd [34]. The intensified values are marginally important for on-site sensing application in which less sophisticated or sensitive apparatuses will be applied for systematic BM analysis [22].

Next, the practical aspect of the presented bioassay was applied for haptoglobin quantification and further compared to the obtained data of conventional bovine haptoglobin ELISA. Corresponding to the enhancement studies, profound haptoglobin content will impede the catalytic process owing to protein–protein (hemoglobin–haptoglobin) complex formation and hence reduce the eCL factor. Indeed, Figure S2 (Supplementary Materials) shows the calibration curve of eCL factor vs. range of haptoglobin concentrations with the addition of cross-linked GNPs, acquired under optimized conditions, that is reduced upon higher target molecule concentrations. The bioassay presented a broad linear response from 1 pg mL^−1^ to 10 µg mL^−1^ and curve equation of eCL factor = (15.562 ± 0.328) − (0.789 ± 0.042) × Ln(C_haptoglobin_). It should be highlighted that the wide range of reference haptoglobin concentrations are prone to heteroscedasticity within semi-logarithmic scale, thus leading to inaccuracy of the calibration [36]. However, herein, the presented correlation coefficient (R^2^ = 0.98) depicts sufficient linearity and minimal statistical error deviation of the sampling points (in the range of 0.4%–5.8%). The bioassay LOD was 0.19 pg mL^−1^ and was calculated using the equation $y_{b}-3Std_{b}$, where $y_{b}$ is the averaged eCL factor measured for the milk-free conditions (maximal optical response without any haptoglobin residuals) and $Std_{b}$ is the associated standard deviation, as previously shown by Krismastuti et al. [37]. The presented LOD intensifies the analytical performance of the current platform with respect to conventional assays and research methods for haptoglobin detection within real samples, see Table S2 for details [38,39,40,41,42,43]. The linear regression was used for calculating haptoglobin concentrations within all analyzed milk samples and the results are summarized in Table 2. The obtained haptoglobin values support the eCL factor studies of the different milk samples, in correlation to the severity of the occurring BM and SCC levels. Both pathogens, *E. coli* and CNS similarly affected by the internal haptoglobin concentration upon similar SCC values, although some exceeding values were shown for SCC >1,000,000 for M3 over M6, 24.0 ± 4.5 and 19.5 ± 3.0 µg mL^−1^ of haptoglobin, respectively. The causative pathogen has been previously shown to influence the inflammatory response and by that inducing the secretion of the target acute phase protein into plasma or milk, resulting in higher response toward *E. coli* over CNS contamination [44]. Finally, the obtained results were compared to bovine ELISA performances and, indeed, similar haptoglobin concentration values were shown for all analyzed samples, both for pathogen types and SCC values. It should be mentioned that the recovery of the resulting values was in the range of 86%–108%, this can be attributed to the different methodologies applied for the sensing assay, colorimetric absorption vs. photon count, while the latter presents enhanced sensitivity. Overall, the developed bioassay of using eCL factor by applying cross-linked GNPs into the luminol assay is adequate for haptoglobin analysis in bovine milk samples. The signal amplification overpowers conventional assay in terms of detection limit, assay time, reduced costs per assay, flexibility, and applicability for on-site detection by simplified portable device. Moreover, it should be highlighted that the presented assay can be personalized and applied for the study of other protein classes upon minor assay modification and improvement.

## 4. Conclusions

In summary, we successfully developed a sensing platform for sensitive haptoglobin detection in bovine milk based on the luminol–H_2_O_2_–hemoglobin CL system for clinical BM status assessment. The obtained haptoglobin levels increased at higher SCC values due to inflammatory reaction to causative pathogen, which inhibit the catalytic activity of the anchored hemoglobin (by complexing into a hemoglobin–haptoglobin scaffold). The addition of optimized cross-linked GNPs (size ~25 nm) produced eCL signal catalysis over 30-fold with respect to nanoparticle-free assay. The studied inherent milk factors, SCC values, and pathogen origin impact the haptoglobin concentrations by modifying eCL intensities. The results reveal a sufficient variation between the different clinical states of the animal in agreement with the existing traditional techniques (ELISA). The presented sensing methodology is an efficient, cost-effective, and label-free technique applicable within the clinical health range for early identification of BM. Moreover, the generic design provides promising approach for rapid and sensitive detection of a wide variety of other acute phase biomarkers for estimating deteriorated clinical status both in humans and animals.

## Figures and Tables

**Table 1:** Somatic cell count (SCC) values and identified pathogen within milk samples.

Sample	SCC	Pathogenic Bacteria
H	60,000	-
M1	300,000	*E. coli*
M2	636,000	*E. coli*
M3	>1,000,000	*E. coli*
M4	337,000	CNS
M5	821,000	CNS
M6	>1,000,000	CNS

Healthy milk sample (H), mastitic milk samples (M). *E. coli*—*Escherichia coli*; CNS—coagulase-negative staphylococci.

**Table 2:** Haptoglobin concentrations with and without cross-linked GNPs in milk samples using eCL factor with respect to bovine ELISA kit.

Sample	Haptoglobin with Cross-Linked GNPs (µg mL^−1^)	Haptoglobin ELISA (µg mL^−1^)
H	0.1 ± 0.1	0.1 ± 0.1
M1	1.4 ± 1.2	1.2 ± 0.01
M2	3.6 ± 1.4	3.9 ± 1.3
M3	24.0 ± 4.5	21.0 ± 0.8
M4	1.6 ± 0.9	1.7 ± 0.3
M5	5.2 ± 1.7	4.8 ± 0.2
M6	19.5 ± 3.0	16.6 ± 3.1

Healthy milk sample (H), mastitic milk samples (M). Data are reported as mean ± standard deviation (*n* ≥ 3).

## Supplementary Materials

The following are available online at https://www.mdpi.com/2218-273X/9/8/372/s1, Table S1: The average diameter of the produced GNPs before and after PDT cross-linking obtained by dynamic light scattering (DLS), Table S2: Analytical performance of optical techniques utilizing nanoparticles for haptoglobin detection, Figure S1: TEM images of the produced GNPs, Figure S2: Calibration curve of eCL factor for standard Hp concentrations with optimal GNPs-PDT.
