# Supplementary material for: Gold Nanoparticle Size-Dependent Enhanced Chemiluminescence for Ultra-Sensitive Haptoglobin Biomarker Detection
Source: Biomolecules. 2019 Aug 14;9(8):372. doi: 10.3390/biom9080372 (PMC6723178; doi:10.3390/biom9080372)
Supplement: Supplementary file 1 [file biomolecules-09-00372-s001.pdf]

Supplementary Materials

Gold Nanoparticle Size-Dependent Enhanced Chemiluminescence for Ultra-Sensitive Haptoglobin Biomarker Detection

Narsingh R. Nirala[1] *and Giorgi Shtenberg*[1]*

[1]Institute of Agricultural Engineering, ARO, the Volcani Center, Bet Dagan 50250, Israel

Corresponding Author

*E-mail: giorgi@agri.gov.il; Tel: +972-50-7795925

Average diameter (nm)		
**Planned**	**Before PDT cross-linking**	**After PDT cross-linking**
2.6	5.2	237.5
8	9.4	63.0
13	10.9	50.7
25	30.5	47.3
38	39.9	68.1

Table S1. The average diameter of the produced GNPs before and after PDT cross-linking obtained by DLS.

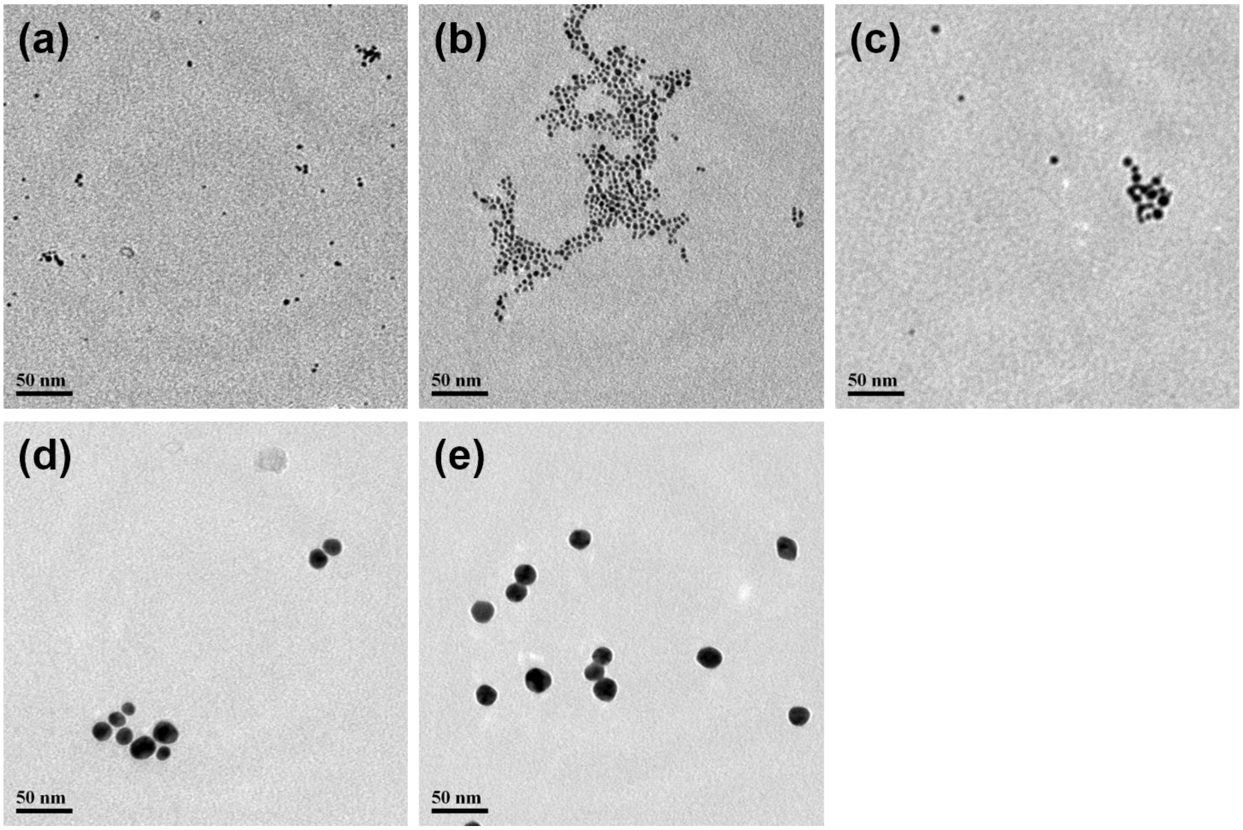

Figure S1. TEM images of the produced GNPs (before PDT cross-linking): (a) 2.6 nm; (b) 8 nm; (c) 13 nm; (d) 25 nm; (e) 38 nm.

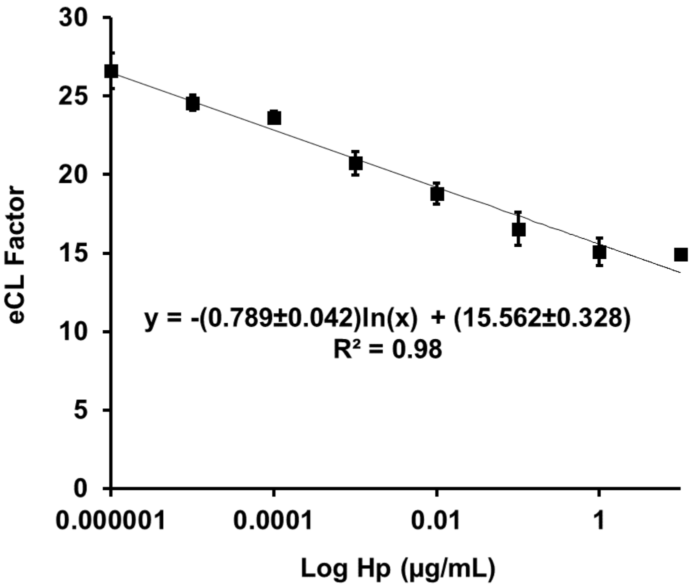

Figure S2. Calibration curve of eCL factor for standard Hp concentrations with optimal GNPs-PDT. Data are reported as mean ± standard deviation (n=3).

Table S2. Analytical performance of optical techniques utilizing nanoparticles for haptoglobin detection

Sensing platform	Optical technique	Dynamic range	Detection Limit	Ref.
Magnetic nanobeads immunoassay	Capillary electrophoretic laser-induced fluorescence	0.2–3.0 mg mL^{-1}	N/A	[1]
CdTe QDs immunoassay	Fluorescence resonance energy transfer	0.1–0.6 nM	0.02nM	[2]
Gold nano layer	Surface plasmon resonance	N/A	1.1 μg mL^{-1}	[3]
Gel electrophoresis	Chemiluminescence	0.1-13.3 μg mL^{-1}	0.08 μg mL^{-1}	[4]
Enzyme-linked immunosorbent assay	Absorbance	N/A	0.01 μg mL^{-1}	[5]
GNPs/CdTe-QDs/SWCNTs/Chitosan nanocomposite	Electro-chemiluminescence	0.1 pg mL^{-1} to 10 ng mL^{-1}	0.1 pg mL^{-1}	[6]
Cross-linked GNPs	Chemiluminescence	1 pg mL^{-1} to 10 μg mL^{-1}	0.19 pg mL^{-1}	This work

References

1. Wang, Y.-R.; Yang, Y.-H.; Lu, C.-Y.; Chen, S.-H. Utilization of magnetic nanobeads for analyzing haptoglobin in human plasma as a marker of Alzheimer's disease by capillary electrophoretic immunoassay with laser-induced fluorescence detection. *Anal. Chim. Acta* **2015**, *865*, 76-82, doi:https://doi.org/10.1016/j.aca.2015.01.030.
2. Abadieh, R.; Safi, S.; Mohsenifar, A.; Bayat, M. Designation of a fluorescence-based nanobiosensor for detection of bovin haptopglobin. *Bull. Georg. Natl. Acad. Sci.* **2015**, *9*, 222-228.
3. Åkerstedt, M.; Björck, L.; Waller, K.P.; Sternesjö, Å. Biosensor assay for determination of haptoglobin in bovine milk. *J. Dairy Res.* **2006**, *73*, 299-305.

4. Huang, G.; Ouyang, J.; Delanghe, J.R.; Baeyens, W.R.G.; Dai, Z. Chemiluminescent Image Detection of Haptoglobin Phenotyping after Polyacrylamide Gel Electrophoresis. *Anal. Chem.* **2004**, *76*, 2997-3004, doi:10.1021/ac035109e.
5. Nakagawa, H.; Yamamoto, O.; Oikawa, S.; Higuchi, H.; Watanabe, A.; Katoh, N. Detection of serum haptoglobin by enzyme-linked immunosorbent assay in cows with fatty liver. *Res. Vet. Sci.* **1997**, *62*, 137-141, doi:https://doi.org/10.1016/S0034-5288(97)90135-1.
6. Rizwan, M.; Keasberry, N.A.; Ahmed, M.U. Efficient double electrochemiluminescence quenching based label-free highly sensitive detection of haptoglobin on a novel nanocomposite modified carbon nanofibers interface. *Sens. Biosensing Res.* **2019**, *24*, 100284, doi:https://doi.org/10.1016/j.sbsr.2019.100284.
